# Identification of a novel *RPGRIP1* mutation in an Iranian family with leber congenital amaurosis by exome sequencing

**DOI:** 10.1111/jcmm.13454

**Published:** 2017-11-29

**Authors:** Saber Imani, Jingliang Cheng, Abdolkarim Mobasher‐Jannat, Chunli Wei, Shangyi Fu, Lisha Yang, Khosrow Jadidi, Mohammad Hossein Khosravi, Saman Mohazzab‐Torabi, Marzieh Dehghan Shasaltaneh, Yumei Li, Rui Chen, Junjiang Fu

**Affiliations:** ^1^ Key Laboratory of Epigenetics and Oncology Research Center for Preclinical Medicine Southwest Medical University Luzhou Sichuan China; ^2^ Hunan Normal University Medical College Changsha Hunan China; ^3^ Chemical Injuries Research Center Baqiyatallah University of Medical Sciences Tehran Iran; ^4^ Student Research Committee Baqiyatallah University of Medical Sciences Tehran Iran; ^5^ The Honors College University of Houston Houston TX USA; ^6^ Department of Molecular and Human Genetics Baylor College of Medicine Houston TX USA; ^7^ Department of Ophthalmology Baqiyatallah University of Medical Sciences Tehran Iran; ^8^ Eye Research Center Farabi Eye Hospital Tehran University of Medical Sciences Tehran Iran; ^9^ Laboratory of Neuro‐organic Chemistry Institute of Biochemistry and Biophysics (IBB) University of Tehran Tehran Iran; ^10^ Laboratory of Systems Biology and Bioinformatics (LBB) Institute of Biochemistry and Biophysics University of Tehran Tehran Iran

**Keywords:** leber congenital amaurosis, *RPGRIP1*, mutation, Iran, target exome sequencing

## Abstract

Leber congenital amaurosis (LCA) is a heterogeneous, early‐onset inherited retinal dystrophy, which is associated with severe visual impairment. We aimed to determine the disease‐causing variants in Iranian LCA and evaluate the clinical implications. Clinically, a possible LCA disease was found through diagnostic imaging, such as fundus photography, autofluorescence and optical coherence tomography. All affected patients showed typical eye symptoms associated with LCA including narrow arterioles, blindness, pigmentary changes and nystagmus. Target exome sequencing was performed to analyse the proband DNA. A homozygous novel c. 2889delT  (p.P963 fs) mutation in the *RPGRIP1* gene was identified, which was likely the deleterious and pathogenic mutation in the proband. Structurally, this mutation lost a retinitis pigmentosa GTPase regulator (RPGR)‐interacting domain at the C‐terminus which most likely impaired stability in the RPGRIP1 with the distribution of polarised proteins in the cilium connecting process. Sanger sequencing showed complete co‐segregation  in this pedigree. This study provides compelling evidence that the c. 2889delT  (p.P963 fs) mutation in the *RPGRIP1* gene works as a pathogenic mutation that contributes to the progression of LCA.

## Introduction

Leber congenital amaurosis  (LCA; MIM # 204000 ) is an autosomal recessive inherited heterogeneous retinopathy, affecting 1 in every 80,000 people worldwide 1, 2. LCA consists of rare early‐onset retinal dystrophies that constitute < 6% of all retinal dystrophies and impact approximately 20% of children attending schools for the blind 3. Severe and early visual loss (typically before the first year of age), an oculo‐digital sign of Franceschetti, sluggish pupillary light reflex, nystagmus, blindness, visual impairment presented in infancy and near‐absent pupillary reflexes are the main clinical symptoms of LCA 4, 5. LCA is characterised by severe  pigmentary degeneration of the fundus, which typically starts in the mid‐periphery and advances towards the fovea and macula 6, 7.

The molecular genetic basis underlying LCA is clinically and genetically heterogeneous; to date, around  22 genes with mutations have been associated  with pathogenesis of LCA 6, 8. These mutations are involved in the various features of the photoreceptors in the outer segment (OS), including development, protein transporting, neurodevelopmental delay, phagocytosis and retinoid cycle 9. Moreover, mutations in the same gene lead to variable phenotypes, adding to the disease's complexity 10.

It is well established that detection of genes related to inherited LCA diseases has triggered major interests in ‘retinopathies’ 11. Increase in patient recruitment to new gene‐ or mutation‐specific trials and overlaps between LCA and other inherited retinal diseases are the main reasons for the misdiagnosis of LCA patients 8, 12. Genetic or molecular methods have already highlighted the diagnostic potential for more than 70% of LCA cases. Furthermore, a noteworthy method to diagnosing the manifestation of this disease only using an electroretinogram (ERG) is insufficient to document the rod and cone responses in  ~50% of LCA patients 13, 14. In this regard, various sequencing methods have contributed to the identification of possible disease‐causing mutations at the whole genome level, each different regarding their turnaround feasibility, customised target capture and time for sequencing of large quantities of data 15.

On the other hand, next‐generation sequencing (NGS) technologies are the most available  and promising methods to identify novel  disease‐causing mutations 15, 16, 17. In the last decade, the use of NGS methods has dramatically increased, including methods like whole genome sequencing (WGS), whole genome re‐sequencing (WGRS), whole exome sequencing (WES) and target exome sequencing (TES), for the identification of genotype–phenotype correlations and novel allele pathogenicity in inherited diseases 18, 19. Furthermore, NGS‐based molecular analysis has been proven to be a strong approach for diagnosis of heterogeneous monogenetic diseases, such as LCA, in a large‐scale level, which can be used towards genetic counselling and potential gene replacement therapy 17, 20, 21, 22.

Here, we employed NGS‐based mutation screening in an autosomal recessive LCA (arLCA) in Iranian family to categorise the potential phenotype–genotype correlation in the affected individuals and to determine the proper impact of genetic factors on disease diversity. These results helped find the particular mutation associated with the disease and revealed the potential characteristics of the LCA mutation spectrum in this population. Our finding provides evidence that a homozygous novel c.2889delT (p.P963 fs) mutation in the retinitis pigmentosa GTPase regulator‐interacting protein 1 (*RPGRIP1*) gene contributes to the progression of causativity and susceptibility variants in LCA patient. We provide genetic and clinical data to support the contention that the recessive mutations in *RPGRIP1* are responsible for arLCA in Iranian family. We have consequently shown this deletion is most likely pathogenic by damaging the RPGRIP1  protein structure in our studied family.

## Materials and methods

### Ethical statement

The research was approved by the Ethical Committees of the *Southwest Medical University*. Written informed consent conforming to the tenets of the Declaration of Helsinki (1983 Revision) 23, 24 was obtained from all participants or their guardians before the study. Prospective volunteers were informed of the purpose and procedure of the study. Also, all clinical assessments were processed according to the local Ethics Committee guidelines of Ophthalmology Center, *Bina Eye* Hospital, Tehran, Iran. The molecular biologists were blinded for all cases.

### Patients and clinical assessment

**Figure 1:**
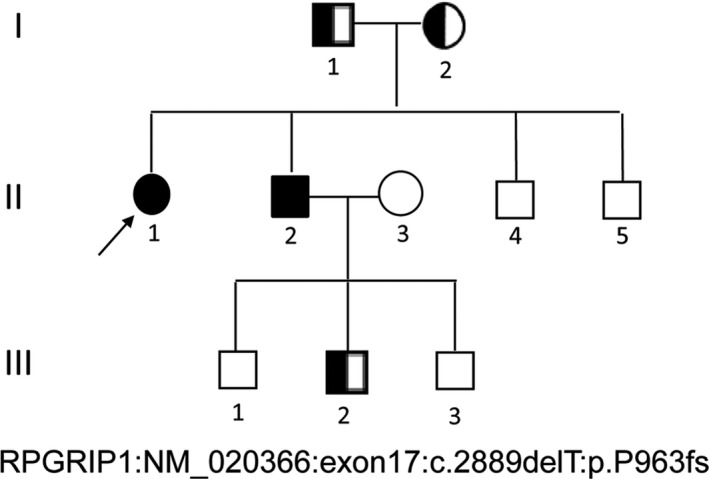
Schematic pedigrees showing in an arLCA family that is described in this study. Family number and disease‐causing mutation are noted in above pedigree. Normal individuals are shown as clear circles (females) and squares (males), affected individuals are shown as filled symbols, and carriers are shown as hemi‐filled symbols. The patient above the arrow indicates the proband (II: 1), where target exome sequencing was performed with deletion mutation of *RPGRIP1*:NM_020366:exon17:c.2889del.T:p.P963 fs.

The studies consisted of two patients including one proband (Fig. 1, pedigree II: 1, arrow), and ten related family members with three generations of familial LCA were recruited in the Tehran, Iran, based on their genetic and pedigree analysis (Fig. 1). The patients were diagnosed with likelihood of LCA by experienced ophthalmologists. Demographic information, inheritance patterns, ethnicity and other personal information, such as age, gender, number of affected patients and members who were accessible for sampling, were documented according to the interviewer‐administered questionnaire . A detailed clinical history and full ophthalmic examinations were performed, including the best‐corrected Snellen visual acuity, Humphrey visual fields,  slit‐lamp biomicroscopy, fundoscopy,  optical coherence tomography (OCT, Carl Zeiss, Oberkochen, Germany), fundus photography (FA, Spectralis; Heidelberg Engineering, Heidelberg, Germany ) and standard electroretinography (ERG, RetiPort ERG System; Roland Consult, Wiesbaden, Germany).

### DNA sampling

Human genomic DNA (gDNA) was extracted from 2 ml of fresh peripheral blood leucocytes using standard Qiagen DNA extraction kit (Qiagen, Hilden, Germany). Blood samples were collected from this pedigree (Fig. 1) in EDTA tubes for DNA extraction. In addition, blood samples were taken from 100 LCA‐unrelated, ethnically matched, healthy control volunteers with no family history of eye disorders. The optical density ratio of 260/280 ~ 1.8 and 260/230 > 1.5 was assayed for gDNA quality and concentration 25. To access the novel LCA disease‐causing genes, we designed the homozygosity genome‐wide sequencing using TES analyses on the DNA sample from the proband.

### Capture panel designing

Here, we used capture agilent probes that were used in previously published studies 21, 26, 27, 28.

### Library preparation and capture sequencing

The design of exome capture panels has been described in previous literature, according to the Illumina paired‐end libraries (Illumina, Inc., San Diego, CA, USA) 21, 26, 27. In brief, 2 μg of extracted gDNA of the proband was randomly sheared into 300–500 bp fragments by sonication. The 5′ ends of all DNA fragments were phosphorylated by polynucleotide kinase, and adenine was added at the 3′ ends. Then, hybridisation to the pre‐capture libraries was quantified by the PicoGreen fluorescence assay kit (Invitrogen, Carlsbad, CA, USA). Each capture reaction and 50 pre‐capture libraries (60 ng/library) were pooled together, and after washing, the panel was recovered using Agilent Hybridization and Wash kit (Agilent Technologies, Santa Clara, CA, USA). Finally, captured DNA libraries were sequenced on Illumina HiSeq 2000 (Illumina, Inc.) at the Baylor College of Medicine core facility, following the manufacturer's protocols.

### Variant filtering and bioinformatic analysis

Paired‐end sequencing illumine reads were aligned to the human hg19 reference genome using Burrows‐Wheeler Aligner version 0.6.1 and available public online UCSC database (http://genome.ucsc.edu/) 29. Single nucleotide polymorphisms (SNPs) and insertions/deletions (INDELs) variations were refined using a toolkit Atlas‐SNP2 and Atlas‐Indel2 (GATK version 1.0.5974) 30. Variant frequency data were applied to online control databases, CHARGE consortium 31, Exome Aggregation Consortium (ExAC), 1000 Genome Project 32, ANNOVAR 33 and ESP‐6500 34 databases, to find the pathogenic mutations in all candidate genes with a minor allele frequency of more than 5%. Sequencing depth 4, estimated copy number 2, SNP quality 20 (score 20 represents 99% accuracy of a base call) and a distance between two SNPs>5 are considered the filtrations criteria for candidate SNPs 35. Because arLCA is a rare Iranian disorder, variants and deep intrinsic exon–intron junctions were filtered out from the following analysis with a frequency higher than 1/400 and distance >10 bp, respectively. Altogether, an average of 3000 SNPs and INDELs was found after applying these filters. Sequence variants were not annotated in any of the above public databases. Consequently, the phenotypes of all cases were similar, so we identified common variants among affected patients (Fig. 1, pedigree II: 1).

### Primer design, PCR amplification and Sanger sequencing

For mutation confirmation and segregation analysis, polymerase chain reaction (PCR) amplification and direct sequencing  of prioritised variants were applied to the gDNA of all the patients. Accordingly, DNA sequences of each identified mutation were obtained from the UCSC Genome Browser. We designed locus‐specific primers using the online Primer3 program (http://primer3.ut.ee/) 36. Then, the PCR products were confirmed by Sanger sequencing methods on an ABI‐3500DX sequencer (Applied Biosystems Inc., Foster City, CA, USA) through the specific primer sequences sorted in Table 1. Finally, the resultant sequences were compared to consensus sequences by Seqman software (Lasergene 8.0; DNASTAR, Inc., Madison, WI, USA). All reactions were performed with two replicates per sample besides a non‐reverse transcription control and non‐template control for each test.

**Table 1:** PCR sequences of *RPGRIP1* primers, product size

Name	Sequence (5′‐3′)	Size (bp)	Tm (°C)
RPGRIP1‐ CII2L	ACTGACCCTGCAGAGAAACC	350	60
RPGRIP1‐ CII2R	ATGTTGGTCAGGCTGGTCTT		

## Results

### Pedigree and clinical phenotypes

**Figure 2:**
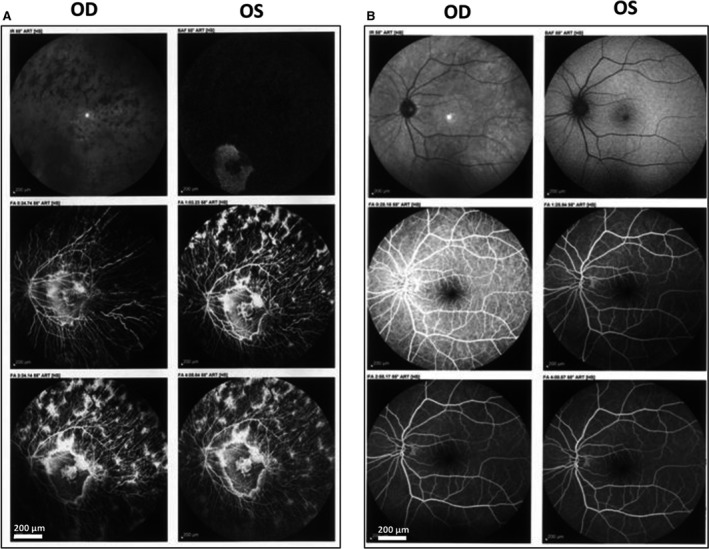
Representative fundus photographs of patient II:1 (proband) from both eyes. Panel **A**.  14‐year‐old Iranian female patient. Panel **B**. Fundus photographs of unaffected age‐matched control. The comparison between two panels clearly has shown the ‘salt and pepper’ pigment mottling pattern, severe RPE atrophic changes and the transparent in the macula in the patient that is afflicted with the disease.

The individual of interest (Fig. 1, II: 1) is a 14‐year‐old Iranian female patient with a probed case of early clinical signs of LCA progression from the age of 4. The lens examination revealed a posterior subcapsular cataract. Best‐corrected visual acuities of the left and right eyes were ranged between 20/100 and 20/30, respectively. Also, the proband showed typical macular atrophy and high myopia. Tunnel vision, decreased night vision and loss of peripheral (side) vision were presented in all affected individuals, mutant homozygous type (pedigree II: 1&2; Fig. 1) and absent in all mutant heterozygous type (pedigree I: 2 & III: 2; Fig. 1). The present symptom for the proband (pedigree II:1) was more severe with night blindness, decreased central vision, visual acuity and especially the presence of fundus flecks in the posterior pole of the retina. The representative fundus photos of the affected individual and normal wild type (Fig. 1, II: 3) are shown in Figure 2. FA results showed the ‘salt and pepper’ pigment mottling pattern, severe retinal pigment epithelium (RPE) atrophic changes, replacement of normal darkened colour with a central reddish colour, and close mottling and transparency of the macula. The FA of the proband showed dark choroid with staining of white fundus flecks (Fig. 2, panel A) in comparison with the eyes of healthy sex‐/age‐matched control (Fig. 2, panel B).

**Figure 3:**
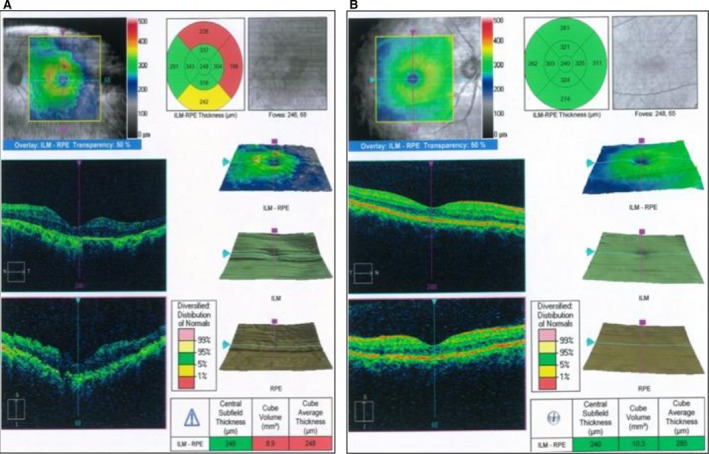
Retinal phenotypes of proband. Panel **A**. Optical coherence tomography and electroretinography features of inherited retinal dystrophies in left eye for II:1. Panel **B**. Representative optical coherence tomography and electroretinography left eye of control. This figure shows that the patient had marked thinning and disruption of the photoreceptor layer, choroid and the retinitis pigment epithelium.

Furthermore, the FA finding was confirmed by OCT and ERG imaging (Fig. 3). Panel A of Figure 3 presents the loss of the outer retinal architecture, foveal atrophy and loss of normal foveal configuration of the proband's left eye, which was extremely apparent in the macula. ERG results showed abnormality in the PRE lyres of proband with cube volume of 8.9 mm^3^ and cube average thickness of 248 μm. In comparison, the cube volume and cube average thickness features of the normal left eye of Iranian age‐matched control were 9.5 mm^3^ and 264 μm (Fig. 3, panel B), respectively, which showed a significant decrease in RPE in the proband case. This figure illustrates a likely disruption of the photoreceptor layer and choroid that is increased by thinning in RPE. To note, macular progressive depigmentation with pigment clumping and atrophy is seen as a major complement to the proband.

### Data sequencing of samples

To access novel LCA disease‐causing genes, targeted capture high‐throughput  sequencing of known RP‐related genes was performed successfully using a custom‐designed capture panel on the gDNA sample of an affected member (Fig. 1, pedigree II: 1). We identified causative mutations in LCA patient by automatic variant calling, filtering and annotation pipeline in the capture sequencing data. The targeted regions with evenness scores more than 0.8 across of all samples were converged. Commonly, 96.0% of the targeted regions have coverage >20× and 91.1% of the targeted regions have coverage >40×. In all, more than 10 million bases of the sequence with 100‐bp read length, 40000 SNPs and 11400 INDELs were generated. After quality assessment, more than 97% of billion bases were aligned to the human reference sequences and, among those, billions of bases covered with a 10‐fold coverage target region. Finally, sequence variants that were not annotated in any of the above public databases were prioritised for further confirmation and characterisation.

### Putative pathogenic mutation screening

Stepwise mutation identification strategy was used to identify pathogenic variants for the included proband 17, 21, 37, 38. We recognised a single nucleotide homozygous deletion (c.2889delT) of *RPGRIP1* gene (NM_020366) in this arLCA, leading to a shift in the reading frame at amino acid position 963 (Fig. 1 with pedigree II: 1). This novel, possibly disease‐causing mutation leads to a big deletion in the C‐terminal of RPGRIP1 protein after amino acid position 963 (p.P963 fs) (more than three‐fourth deletion of RPGRIP1), due to a frameshift resulting in 36 incorrect amino acids after codon 962, followed by premature termination at codon 999 (p.P963 fs*999).

### Mutation validation and segregation analysis

**Figure 4:**
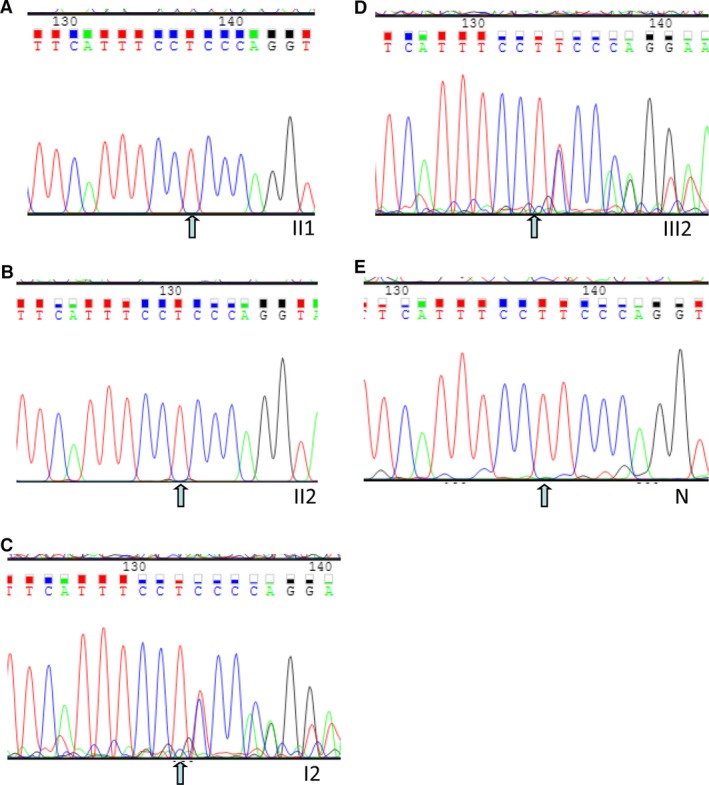
Sanger sequencing validation. **A, B, C, D** and **E** indicate the sequencing results in II: 1, II: 2 (mutant homozygous type), I: 2, III: 2 (heterozygous type), N (wild type, normal control: a normal person from no eye disease history family), respectively. The arrows indicate the deletion at the nucleotide position NM_020366:exon17:c.2889del.T in *RPGRIP1* gene.

Albeit deficient, the Sanger sequencing was used for confirmation c.2889delT variant of *RPGRIP1* (Fig. 4). The c.2889delT variant was confirmed in the mutant homozygous type patients (pedigree II: 1&2; Fig. 4A and B) and mutant heterozygous type or carriers (pedigree I: 2 & III: 2; Fig. 4C and D) in the family. The c.2889delT variant was co‐segregated with the disease phenotype in this family's members. Figure 4E shows the representative Sanger sequencing for *RPGRIP1*: NM_020366:exon17: c.2889delT; this mutant is absent in unaffected family individuals and unrelated 100 normal controls, including those without a family history of eye disease (wild type, depicted in Fig. 1 with pedigree III:2). These findings show complete co‐segregation  in the pedigree for the arLCA family and pinpoint its role in LCA pathogenesis.

### Functional effects of  pathogenic mutation

**Figure 5:**
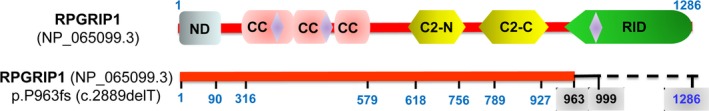
Schematic diagram of the alignment scores for *RPGRIP1* SNPs and protein amino acid residues, corresponding to the c.2889del.T:p.P963 fs. The highlighted amino acid residues in blue are conserved the same. The mutation position is shown in the grey box. The red line shows the alignment scores ≥200, the tiny black line shows the alignment scores ≤40, and the dashed line shows the totally deletion part, between the query of the mutations and wild type. ND, nuclear domain; C2‐N, N terminal of protein kinase C conserved domain 2; C2‐C, C‐terminal of protein kinase C conserved domain 2; CC, coiled‐coil domain; RID, RPGR‐interacting domain.

The overall alignment scores for *RPGRIP1* SNPs and protein amino acid (aa) residues, corresponding to the c.2889delT (p.P963 fs), are shown in Figure 5. Fewer alignment scores of ≤40 aa were in the deletion domain of the RPGRIP1, ranging within acid amine 963–999. This novel, possibly disease‐causing mutation, leads to more than three‐fourth deletion in the C‐terminal of RPGRIP1 protein after amino acid positions 963 (p.P963 fs). This domain is revealed to be the cognate RPGR‐interacting domain (RID). Thus, this figure clearly shows that p.P963 fs made the pathogenic deletion in the C‐terminal of the RPGRIP1 protein. The results point to the probability that p.P963 fs  frameshift mutation most likely leads to a larger, structurally abnormal, unstable and certainly functional RPGRIP1  in the RID domain 14.

The deleterious and pathogenic aspects of c.2889delT (p.P963 fs) mutation are presented in Table 2. The damaging consequence of protein function analysis indicates that the variant in *RPGRIP1* gene was probably the ‘disease‐causing’ and damaging mutation in the Iranian arLCA family. Comprehensively, recessive RPGRIP1 homozygous mutations, c.2889delT (p.P963 fs) mutation, cause  severe LCA in this study. 

**Table 2:** Protein structure and disease‐causing effects of RPGRIP1  SNPs in Iranian arLCA family

Exon	Variation				EXAC
	Nucleotide*	Protein*	Type	Status	
17	c. 2889del.T	p.P963 fs	Deletion	Homo	Novel

c, variation at cDNA level; p, variation at protein level; Homo, homozygote; ExAC, Exome Aggregation Consortium.  *All nucleotide and amino acid abbreviated according to the International Union of Pure and Applied Chemistry   (IUPAC).

## Discussion

Our study presented the phenotypic variety of an affected Iranian proband in arLCA family with a novel c.2889delT (p.P963 fs) *RPGRIP1* homozygous mutation. These findings highlighted that the variant in the *RPGRIP1* gene is likely the deleterious and disease‐causing mutation in this family, thereby expanding the *RPGRIP1* mutation spectrum for arLCA.

**Figure 6:**
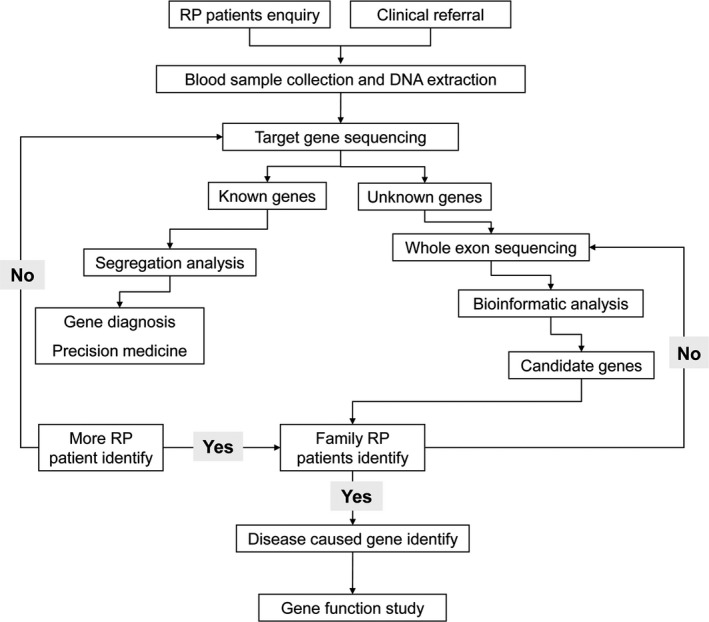
Flow chart showing an approach of this kind of study. Flow chart for genetic tests, selection of patients and WES system in the Iranian LCA family that was described in this study.

High rate of consanguineous marriages and the increase in incidence of LCA in Iran provide a correct platform to study gene mutations that cause LCA 39, 40. Figure 6 proposes an approach used in our group 21, 26, 27; this flow chart gives an overview of the steps followed in predicting the rise of the novel disease‐causing mutations according to NGS‐based models 20. This flow chart is designed for genotype–phenotype descriptions and characterisation of any RP‐related disease, like this arLCA family, using a systematic approach to sequence a set of novel disease‐causing genes. According to this diagram, the phenotypic features of our studied family included bilateral LCA, each in an affected sibling. Several studies have shown that various types of *RPGRIP1* mutations have contributed to nearly two‐thirds of an autosomal recessive cone‐rod dystrophy and some forms of macular dystrophy disease 8, 11, 41.

The *RPGRIP1* gene (NM #020366, OMIM #610937; similarly termed as CORD13, LCA6, RGI1 and RGRIP) evolutionarily encodes retinitis pigmentosa GTPase regulator‐interacting protein 1 (RPGRIP1), with 1286 amino acids in length and a predicted molecular weight of 144 kD 42, 43. The *RPGRIP1* sequences have highly conserved guanine nucleotide exchange factors that interact with retinitis pigmentosa GTPase regulator (RPGR) 11. Functionally, *RPGRIP1* is involved in many biological functions, such as nuclear localisation, neural precursor cell proliferation, retina development in the camera‐type eye, disc morphogenesis, gene expression regulating and disc morphogenesis 42. The expression levels of *RPGRIP1* are greatly reduced in amacrine neurons and rod photoreceptors cells 10, 44. The RPGRIP1, as a scaffolding protein, is required for the survival of photoreceptor cells with the normal location of RPGR at the connecting cilium of photoreceptors’ OS in the retina 6, 43. It is well established that mutations in the *RPGRIP1* gene are associated with cilium dysfunctional syndromes, such as Joubert syndrome (MIM #213300) 45, Leber's hereditary optic neuropathy (LHON, MIM #535000) 46 and Meckel syndrome (MIM #249000) 1, 47. Our literature review shows that mutations in *RPGRIP1* are responsible for more than 5% of type 6 LCA disease (LCA6, MIM #605446) 11, 44. However, the full frequency spectrum of variation in this gene has not been estimated in most Asian countries. The LCA6 is caused by mutations that affect genes represented in this study. It has been found that RPGRIP1 serves as a scaffold to anchor regulatory complexes, including RPGR within the connecting cilium in LCA6 patients. Eventually, mutations in *RPGRIP1* lead to decreased visual acuity, sensitivity in the central visual field and normal fundus findings at birth followed by salt and pepper appearance of FA 6, 9, 40, 43.

In this family, we found a novel deletion in the C‐terminal of RPGRIP1 protein. The homology finding shows that this region of RPGRIP1 is highly conserved and predicted a globular domain that is involved in membrane and vesicular trafficking. This domain is revealed in the cognate RID of C‐terminus in RPGR‐interacting domain (Fig. 5) 43. The absence of exon 17 would cause a misreading of p.P963 fs frameshift that is estimated to lead to the loss  of whole RID domain in more than three‐fourths of the RPGRIP1 protein. Functionally, with any deletions in  the RID, the translocation of RPGRIP1 protein to the proteolytic cleavage and nucleus of the N‐ terminal domain will fail.  Recently, studies have also suggested that *RPGRIP1* is an essential factor in maintaining polarised protein distribution and cilium connection by restricting redistribution and/or directional transport 42, 43.

LCA6 mutations were mapped in Asian LCA family, such as Chinese 40, 48, Indian 49, Turkish 50 and Pakistani 51, according to the identification of *RPGRIP1*  mutations. For example, in the series of *Khan et al*. study, *RPGRIP1* gene accounted for 9% of LCA6 cases in Saudi Arabian families 44. *Dryja et al*. screened seven LCA causative genes in 57 unrelated LCA6 cases and found *RPGRIP1* mutations in 6% of the patients 42. In another genetic study, 5.6% of homozygous nonsense mutations for *RPGRIP1* were found in two consanguineous families with LCA6 52. Our patients presented severe phenotypes of LCA (which may be LCA6), with no light perception and fundus findings ranging from maculopathy to diffuse pigmentary retinopathy 48, 49, 50, 51, 52. Overall, our finding will aid in identifying the particular mutations that affect the molecular pathways  of the LCA patients, which would support the development of disease‐causing gene replacement.  This approach observed any possible correlations among genes investigated in all 18 types of LCA and avoided the unnecessary exclusion of the candidates (Fig. 6).  Although these GWAS and WES studies defined *RPGRIP1* gene as a remarkable candidate in LCA6, the interactions between RPGRIP1 experiences in the RID and RPGRIP1′s vital function for the survival of photoreceptor cells affected those mutations are quite obscure in *RPGRIP1*‐related LCA 20. With the help of numerous genetic studies, the identification and characterisation of this novel mutation in LCA extend the mutation spectrum of *RPGRIP1* gene. Furthermore, definite functional consequences of this mutation and cloning of new candidate genes for LCA and RP patients are required.

To the best of our knowledge, this is the first report that applied TES‐based comprehensive genetic evaluation of LCA variations in Iranian patients, supporting the evidence that this mutation contributes to the causative or susceptible variant in a more severe form of LCA. Our study extends the mutation spectrum of *RPGRIP1* gene and confirms the genotype–phenotype relationship, which may be helpful in genetic counselling and medical managements for LCA patients in development countries, including in Iran.

## Conflict of interest

The authors declare no conflict of interest.

## Author contributions

J.F. and R.C. were in charge of idea, project design and concept of the paper. J.C., C.W. and L.Y. performed PCR amplification, Sanger sequencing and data analysis. Y.L. and R.C. did experiment of NGS and analysed data. A.M.J. and M.H.K. recruited the clinical samples and DNA extraction. K.J. and S.M.T. were in charge of clinical assays. S.I. and M.D.S. performed bioinformatic analysis. J. F., S.F. and S.I. wrote, edited and revised the manuscript. All authors read and approved the manuscript.
